# Immune imprinting and antibody profiles to SARS-CoV-2 in urban and rural Ghana

**DOI:** 10.1016/j.isci.2025.112511

**Published:** 2025-04-23

**Authors:** Martin Montiel-Ruiz, Elvis S. Lomotey, Elizabeth Obeng-Aboagye, Isaac Quaye, Daniel A. Odumang, Florence B. Amakye, Bernard A. Logonia, Salomé Lochmann, Joseph A. Hayford, Dickson K. Osabutey, Angelica Daakyire, Christopher Dorcoo, Edward Dumashie, Joseph Quartey, Dorothy Yeboah-Manu, George B. Sigal, Scott D. Boyd, Irene Owusu Donkor, Katharina Röltgen

**Affiliations:** 1 Department of Medical Parasitology and Infection Biology, Swiss Tropical and Public Health Institute, Allschwil, Switzerland; 2 University of Basel, Basel, Switzerland; 3 Department of Epidemiology, Noguchi Memorial Institute for Medical Research, University of Ghana, Legon, Accra, Ghana; 4 West African Centre for Cell Biology of Infectious Pathogens, College of Basic and Applied Sciences, University of Ghana, Legon, Accra, Ghana; 5 Department of Parasitology, Noguchi Memorial Institute for Medical Research, University of Ghana, Legon, Accra, Ghana; 6 Noguchi Memorial Institute for Medical Research, University of Ghana, Legon, Accra, Ghana; 7 Meso Scale Diagnostics, LLC., Rockville, MD, USA; 8 Department of Pathology, Stanford University, Stanford, CA, USA; 9 Sean N. Parker Center for Allergy and Asthma Research, Stanford University, Stanford, CA, USA

**Keywords:** Health sciences, Medicine, Medical specialty, Immunology, Public health

## Abstract

Infections with SARS-CoV-2 and the development of immunity to the virus and its antigenic variants have been unevenly documented globally, with African populations particularly understudied. As SARS-CoV-2 transitions toward being an endemic pathogen, a more nuanced understanding of immune protection in diverse populations is required. In 2023, we conducted a cross-sectional study of 1,000 Ghanaian residents to assess SARS-CoV-2 prevalence and antibody correlates of immunity against SARS-CoV-2 variants. We found an active SARS-CoV-2 infection rate of 1.3% and a 57% vaccination rate. We observed anti-SARS-CoV-2 Spike plasma antibody sero-positivity of 98.7%, with urban compared to rural residents having higher anti-SARS-CoV-2 plasma and saliva antibody concentrations. Vaccinated and urban individuals exhibited significantly greater Spike-pseudotyped virus neutralization than nonvaccinated and rural individuals. Notably, plasma antibodies preferentially bound Wuhan-Hu-1 over Omicron Spike variants. Our findings indicate significant prior and ongoing SARS-CoV-2 transmission as well as immunological imprinting by Wuhan-Hu-1-like SARS-CoV-2 in Ghana.

## Highlights

• SARS-CoV-2 prevalence and immunity assessed in rural and urban Ghana
• High levels of prior and ongoing SARS-CoV-2 transmission observed
• Urban, vaccinated individuals show greater Spike-pseudotyped virus neutralization
• Significant immunological imprinting by Wuhan-Hu-1-like SARS-CoV-2 detected

## Graphical abstract

**Figure:**
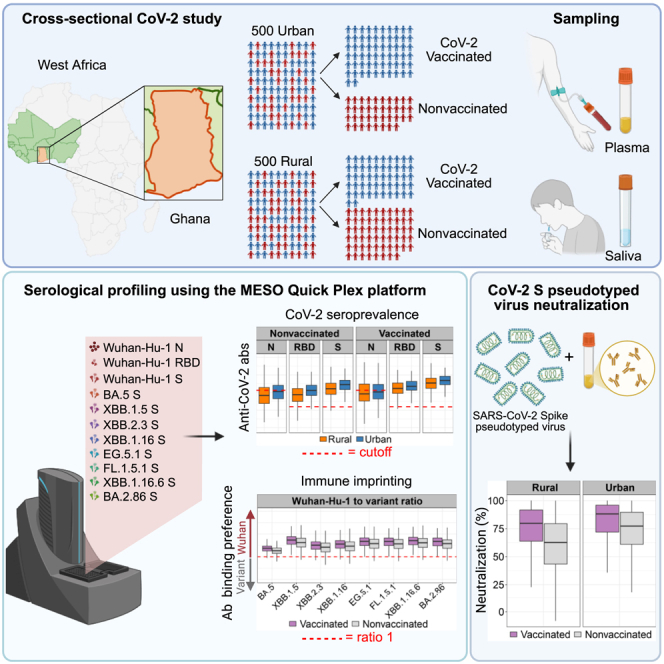

## Introduction

Many African countries reported significantly lower SARS-CoV-2 (CoV-2) case numbers and COVID-19-associated hospitalizations and deaths compared to countries on other continents (https://coronavirus.jhu.edu/map.html). Factors contributing to the observed discrepancies have not been determined with certainty, but could include the younger population age structure, lower levels of urbanization in Africa, host genetic factors, pre-existing cross-reactive immunity, and/or more effective immune responses to CoV-2 infection. Underreporting of hospitalizations and deaths, combined with very limited diagnostic testing, could also play an important role. In fact, sero-epidemiological investigations conducted across African countries indicate infection rates comparable to, or even surpassing, countries in other global regions.^1^^,^^2^ True numbers of COVID-19-related deaths in Africa are difficult to ascertain, considering local limitations around mortality registration and the unavailability of reliable all-cause mortality data for excess death estimations.^3^^,^^4^ It is therefore still unclear whether the impact of COVID-19 in Africa compared to other continents has actually been substantially lower or has just been vastly underestimated.^5^ As CoV-2 has transitioned toward being an endemic virus, research efforts to unravel the apparent riddle of the disease in Africa have further declined. Low vaccination rates in many African countries (https://africacdc.org/covid-19/covid-19-vaccination/) may render populations vulnerable to new CoV-2 infection waves in the future, in particular because CoV-2 variant-updated vaccines have not been available.

Understanding of systemic and mucosal immune responses of African populations to CoV-2 infection and vaccination is similarly impaired by a lack of data, particularly from West, Central, and East Africa. Geographical variation in population responses to vaccines targeting various pathogens is increasingly being recognized and has been linked to genetic factors and environmental exposures.^6^ Differences in microbiota composition, dietary nutrition, and exposures to antigenically similar pathogens causing immunological imprinting, or antigenically divergent pathogens triggering trained innate immunity, have been implicated in vaccine responses.^6^^,^^7^^,^^8^ Efficacy and immunogenicity of different vaccines were not only lower in low- and middle-income compared to high-income regions,^6^^,^^9^^,^^10^^,^^11^ but also in poor rural areas compared to affluent urban regions within the same country.^12^^,^^13^ Studying immune responses to COVID-19 vaccines in understudied African populations is essential to optimize vaccination strategies and other preventive measures in these disadvantaged populations. Geographical variation in immune responses to viral infection is even less well characterized due to the limited availability of data from specific regions of interest. The high CoV-2 infection rates across global populations present a valuable opportunity to collect and compare immunological data from diverse populations.

Early COVID-19 studies mainly conducted in the Americas, Europe, and South Africa have revealed important features of adaptive immune responses to CoV-2.^14^^,^^15^ Infection with the virus elicits antibody responses to the Spike surface protein, its receptor-binding domain (RBD), and the immunodominant Nucleocapsid (N) protein in most patients.^16^^,^^17^^,^^18^ Antibodies against RBD, and to a lower extent against other Spike epitopes, can neutralize the virus by preventing its interaction with the host cell.^19^ The Spike protein is the sole viral target of many COVID-19 vaccines, including the adenoviral AstraZeneca and Johnson&Johnson vaccines and mRNA-lipid nanoparticle vaccines produced by Pfizer-BioNTech and Moderna. These vaccines have demonstrated high safety and efficacy for the prevention of severe COVID-19 and death,^20^^,^^21^^,^^22^^,^^23^ with concentrations of immunoglobulin G (IgG) against Spike or RBD and virus neutralizing antibody titers identified as the best correlates of protection.^24^^,^^25^^,^^26^ CoV-2 variants have emerged that evade immunity developed against early Wuhan-Hu-1-like CoV-2.^27^^,^^28^^,^^29^ Notably, a bias toward the immune recognition of the first CoV-2 variant encountered by an individual, and decreased responses to subsequent viral variants has been reported,^30^ a phenomenon referred to as “immunological imprinting.”^31^^,^^32^ Mechanistically, imprinting is likely caused by the formation of memory B cell populations during initial viral exposures that then influence which epitopes of subsequent viral variants will give rise to dominant responses.^33^^,^^34^^,^^35^

To begin to address knowledge gaps concerning the immune responses to CoV-2 infection and vaccination in African populations, we studied urban and rural communities in the Greater Accra and Eastern Regions of Ghana. West African countries, including Ghana, have been exposed to major global CoV-2 variants, including Alpha, Beta, Gamma, Delta, Eta, Kappa, and Omicron.^36^ At the time of our study, Ghana had reported only 1,462 COVID-19-related deaths and a relatively low rate of 37% fully vaccinated individuals (https://www.ghs.gov.gh/covid19/), with no availability of variant-updated vaccines. To study anti-CoV-2 systemic and mucosal antibody responses in Ghana and go beyond merely reporting seroprevalence rates, we analyzed antibody concentrations to a broad panel of CoV-2 (variant) antigens using multiplexed electrochemiluminescence (ECL) assays and CoV-2 Spike pseudotyped virus neutralization assays. In addition, we measured CoV-2 N antigen in saliva to detect active infections. We identified a high prevalence of previous and current CoV-2 infections in rural and urban Ghana, with significantly higher anti-CoV-2 plasma and saliva antibody concentrations in urban populations. Vaccinated residents exhibited significantly greater neutralization activity against CoV-2 Spike pseudotyped viruses than nonvaccinated people. Notably, we found a striking bias of antibody binding to Wuhan-Hu-1-like CoV-2 variants to the detriment of Omicron recognition.

## Results

### Demographics of the study population and COVID-19-related data

**Figure 1:**
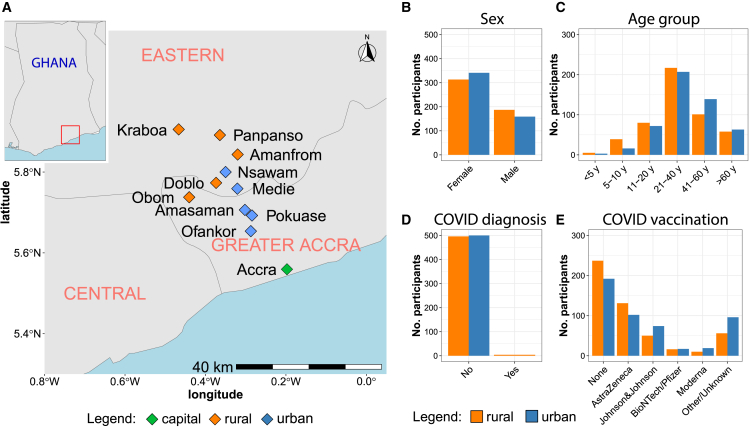
Study site and demographics of study participants Map of Ghana depicting rural (orange) and urban (blue) communities sampled (A). Number of study participants stratified by sex (B), age group (C), previous COVID-19 diagnosis (D), and type of COVID-19 vaccination received (E).

We enrolled 1,000 volunteers, 100 in each of five rural and five urban communities in the Greater Accra and Eastern Region of Ghana (Figure 1A). Selected urban settlements are situated along the main highway between Accra and Kumasi and have a higher population density (greater than 5,000 individuals) and better infrastructure compared to rural areas. In contrast, rural communities are remote settlements with a scattered distribution of the population (less than 5,000 individuals) and limited access to infrastructure, typically only reachable via unpaved gravel or earth roads. Of the 500 rural residents, 187 were male and 313 were female (63%), with an age range between three and 83 years old. Of the 500 urban residents, 159 were male and 341 were female (68%), with ages ranging from three to 90 years old (Figures 1B and 1C). Only four of the 1,000 study participants reported having had a positive CoV-2 test in the past (Figure 1D). The COVID-19 vaccination rate was 62% in urban and 52% in rural communities, with most of the study participants vaccinated with adenoviral-vectored vaccines encoding the Wuhan-Hu-1 CoV-2 Spike protein (AstraZeneca or Johnson&Johnson) and fewer with Wuhan-Hu-1 Spike mRNA vaccines (PfizerBioNTech or Moderna) (Figure 1E). Vaccinated study participants received one (35%), two (44%), or three (21%) vaccine doses. None of the study participants received CoV-2 variant-updated vaccines.

### High levels of CoV-2 exposure in rural and urban areas of Ghana

**Figure 2:**
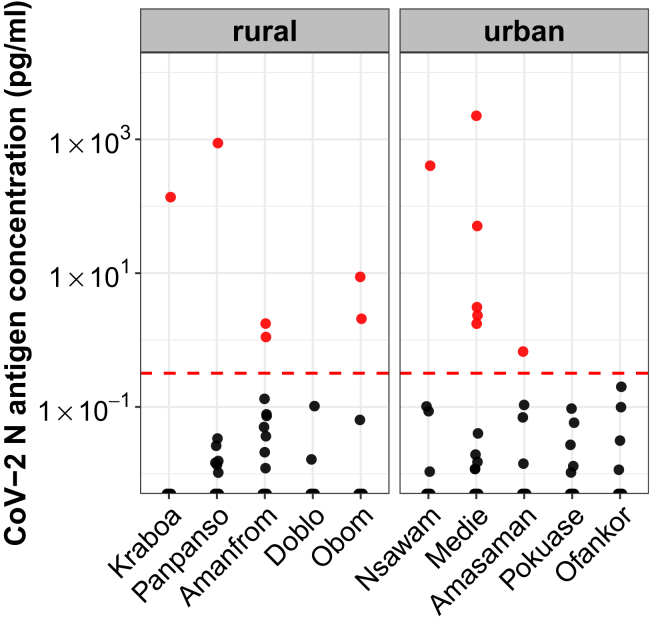
Nucleocapsid (N) antigen detection in saliva as a marker of CoV-2 infection N antigen was detected in saliva using MSD electrochemiluminescence (ECL)-based immunoassays. N antigen concentrations from samples collected in rural and urban communities are shown in red (positive signal) and black (below cutoff for positive). The red dashed line indicates the threshold for positive (0.32 pg/mL) as determined in.^37^

To estimate the prevalence of CoV-2 infection in the Ghanaian study population at the time point of sample collection in November and December 2023, we tested saliva samples from 992 study participants for the presence of the CoV-2 N antigen using an ultrasensitive S-Plex CoV-2 N electrochemiluminescence immunoassay (Meso Scale Discovery, MSD). In previous studies, this assay was found to be highly accurate with a clinical sensitivity of 0.87 (95%CI, 0.83–0.90), a specificity of 0.92 (95%CI, 0.89–0.95),^37^ and an analytical sensitivity of <0.32 pg/mL.^38^ Here, we determined an N antigen positivity rate of 1.3%, indicating active CoV-2 infection in six individuals from rural and seven individuals from urban communities (Figure 2). Of the 13 N antigen-positive individuals, ten had received one or two doses of COVID-19 vaccines. Six were asymptomatic, one reported to have had a cough, two had a fever and/or chills, six had body and/or muscle aches, and five had headaches. Notably, none of these individuals reported shortness of breath, sore throat, or loss of taste or smell.

**Figure 3:**
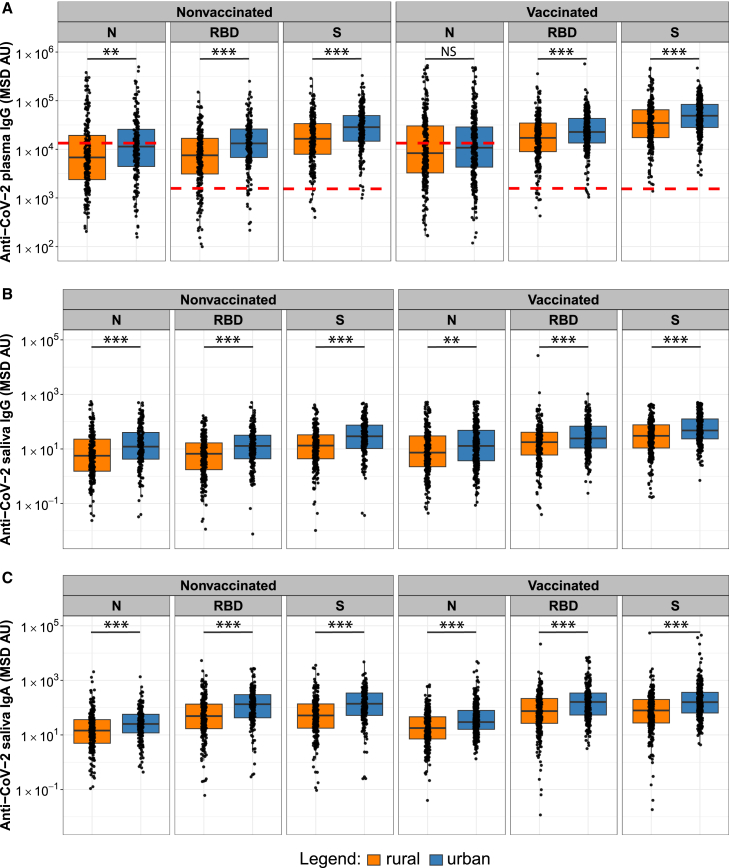
Exposure of rural and urban populations to CoV-2 Graphs show anti-N, anti-RBD, and anti-Spike (S) plasma IgG (A), saliva IgG (B), and saliva IgA (C) antibody concentrations in MSD Arbitrary Units (AU) for nonvaccinated and vaccinated rural (orange) and urban (blue) populations. Box-whisker plots show the median and interquartile range as the box and the whisker ends as the most extreme values within 1.5 times the interquartile range below the 25% quantile and above the 75% quantile. Red dashed lines indicate the cutoff values for the positivity of each assay based on antibody concentrations determined for plasma samples collected before the pandemic started. Significance between rural and urban groups was tested with two-sided Wilcoxon rank-sum test. ∗*p* < 0.05, ∗∗*p* < 0.01, and ∗∗∗*p* < 0.001.

To assess previous exposure of the rural and urban study population to CoV-2 via infection or vaccination, we measured anti-CoV-2 IgG antibody concentrations in plasma samples from 989 study participants. We determined negative cutoff values by calculating mean anti-CoV-2 IgG antibody concentrations plus three standard deviations for plasma samples collected in Ghana before the pandemic started (for details, see STAR Methods section). We found that 98.7% of the 989 participants had been exposed to the CoV-2 Spike protein (Figure 3A). A few of the pre-pandemic plasma samples collected from Ghanaians before the pandemic started had relatively high anti-CoV-2 N IgG concentrations. The high cutoff value determined for the N antigen from this pre-pandemic sample set is likely to underestimate anti-N antibody positivity (only 40% of the study participants had antibody concentrations above the threshold) (Figure 3A).

We found significantly higher levels of anti-N (*p* < 0.01), anti-RBD (*p* < 0.001), and anti-Spike (*p* < 0.001) plasma IgG (Figure 3A) in nonvaccinated urban compared to nonvaccinated rural populations, likely reflecting higher levels of infection with CoV-2 in urban compared to rural settings. Among vaccinated individuals, urban compared to rural populations had significantly higher anti-RBD (*p* < 0.001) and anti-Spike (*p* < 0.001) IgG antibody concentrations, while no significant difference was observed for anti-N plasma IgG (Figure 3A), indicating higher responses to COVID-19 vaccines in urban populations. Notably, we found significantly higher anti-N, anti-RBD, and anti-Spike saliva IgG (Figure 3B) and IgA (Figure 3C) antibody concentrations in urban compared to rural populations irrespective of vaccination status (*p* < 0.001 for all test, except for anti-N saliva IgG in vaccinated populations, where *p* < 0.01). It has previously been reported that saliva IgA levels are relatively short-lived and reach baseline levels about three months after infection.^39^^,^^40^ High anti-CoV-2 saliva IgA concentrations observed particularly in our urban study population indicate high levels of relatively recent CoV-2 infection.

Both urban female and male study participants exhibited higher anti-N, anti-RBD, and anti-Spike plasma IgG (Figure S1A), saliva IgG (Figure S1B), and saliva IgA (Figure S1C) compared to their rural counterparts, regardless of vaccination status. We also detected variations in median plasma IgG (Figure S2A), saliva IgG (Figure S2B), and saliva IgA (Figure S2C) antibody concentrations in residents of different communities and observed higher anti-N, anti-RBD, and anti-Spike plasma IgG (Figure S3A), saliva IgG (Figure S3B), and saliva IgA (Figure S3C) antibody concentrations in nonvaccinated urban compared to rural populations across different age groups. Mean anti-CoV-2 saliva IgA antibodies showed a trend toward increased concentrations in communities located in closer proximity to Accra (Figure S2C). Overall, mean antibody concentrations were strikingly similar in different age groups (Figure S3).

### Impact of COVID-19 vaccination status on systemic and mucosal antibody responses

**Figure 4:**
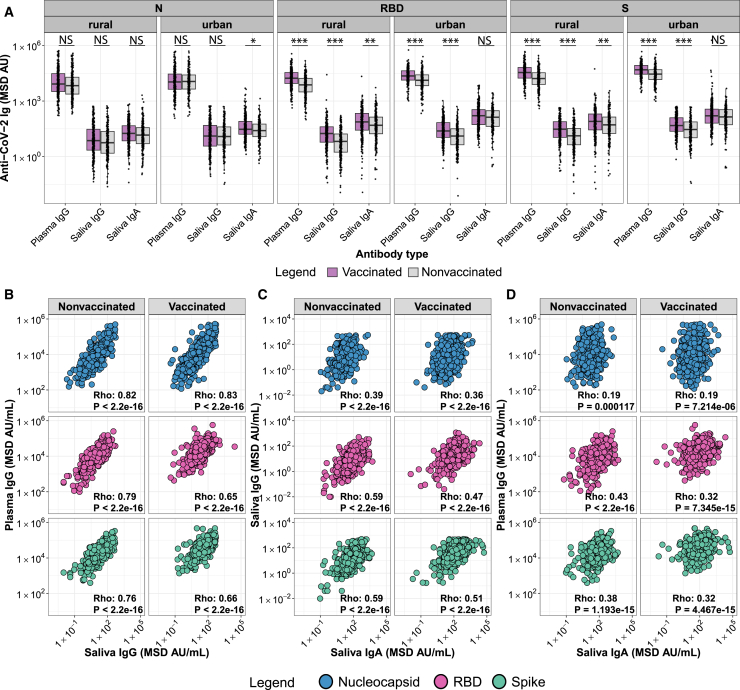
Impact of COVID-19 vaccination status on plasma and saliva antibodies Anti-N, anti-RBD, and anti-Spike plasma and saliva antibody concentrations in MSD Arbitrary Units (AU) are depicted for nonvaccinated (gray) and vaccinated (purple) rural and urban populations. Box-whisker plots show the median and interquartile range as the box and the whisker ends as the most extreme values within 1.5 times the interquartile range below the 25% quantile and above the 75% quantile. Significance between nonvaccinated and vaccinated groups was tested with two-sided Wilcoxon rank-sum test. ∗*p* < 0.05, ∗∗*p* < 0.01, ∗∗∗*p* < 0.001 (A). Correlation of anti-SARS-CoV-2 N (blue), RBD (pink), and Spike (green) plasma IgG versus saliva IgG (B), saliva IgG versus saliva IgA (C), and plasma IgG versus saliva IgA (D) are shown for nonvaccinated and vaccinated populations. Spearman rank correlation (coefficient = Rho, displayed in the plot for each comparison) was used to assess the strength of correlation.

To evaluate the impact of COVID-19 vaccination on antibody concentrations to CoV-2 antigens in the Ghanaian population, we compared anti-N, anti-RBD, and anti-Spike plasma IgG and saliva IgG and IgA between nonvaccinated and vaccinated urban and rural populations. Overall, median anti-N, anti-RBD, and anti-Spike plasma IgG antibody concentrations were around three orders of magnitude higher than saliva IgG concentrations (Figure 4A). We observed higher anti-RBD and anti-Spike plasma and saliva IgG (*p* < 0.001 for all tests) and saliva IgA (*p* < 0.01 for rural participants) antibody concentrations in vaccinated compared to nonvaccinated populations. No significant differences were observed for anti-N plasma and saliva IgG antibody concentrations across populations, but slightly higher saliva IgA (*p* < 0.05) was detected in vaccinated compared to nonvaccinated urban populations.

While plasma and saliva IgG antibody concentrations showed a strong Spearman rank correlation for all antigens (Figure 4B), saliva and plasma IgG compared to saliva IgA antibody concentrations were less well correlated (Figures 4C and 4D). Comparison of Spearman coefficients shows stronger correlation of all antibody combinations against the RBD and Spike antigens in nonvaccinated compared to vaccinated individuals (Figures 4B–4D).

### Increased virus neutralization activity in urban and vaccinated individuals

**Figure 5:**
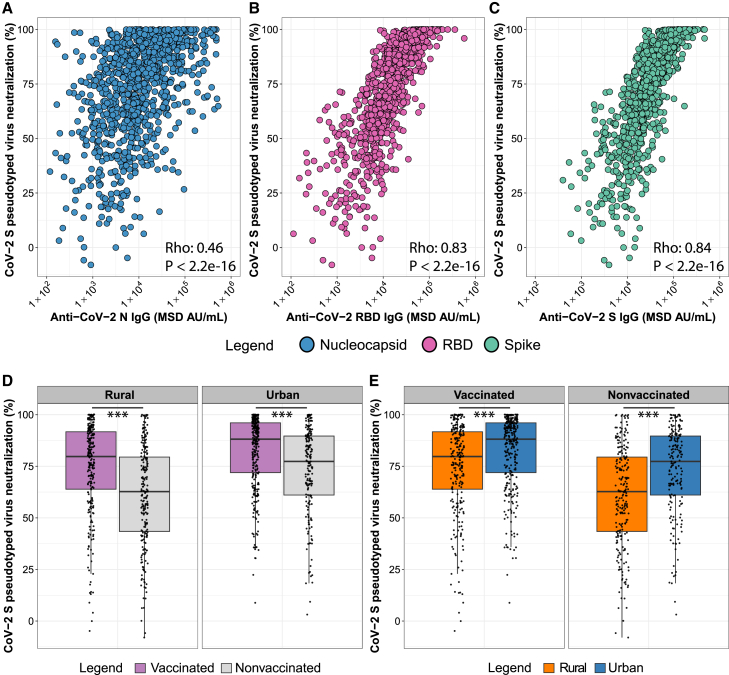
CoV-2 Spike pseudotyped virus neutralization by the plasma samples Correlations between CoV-2 Spike pseudotyped VSV neutralization activity and anti-N (A), anti-RBD (B), and anti-Spike (C) plasma antibody concentrations in MSD Arbitrary Units (AU) are shown. Spearman rank correlation (coefficient = Rho, displayed in the plot for each comparison) was used to assess the strength of correlation. CoV-2 Spike pseudotyped VSV neutralization activity is depicted (as percent neutralization) for nonvaccinated (gray) and vaccinated (purple) (D) as well as for rural (orange) and urban (blue) (E) populations. Box-whisker plots show the median and interquartile range as the box and the whisker ends as the most extreme values within 1.5 times the interquartile range below the 25% quantile and above the 75% quantile. Significance between nonvaccinated and vaccinated groups was tested with two-sided Wilcoxon rank-sum test. ∗*p* < 0.05, ∗∗*p* < 0.01, ∗∗∗*p* < 0.001.

For a functional assessment of the plasma antibodies to prevent viral entry into cells, we also analyzed Wuhan-Hu-1 CoV-2 Spike pseudotyped vesicular stomatitis virus (VSV) neutralization activity by the 989 plasma samples and evaluated correlations with the anti-N (Figure 5A), anti-RBD (Figure 5B), and anti-Spike (Figure 5C) plasma IgG binding concentrations. Neutralization showed strong correlations with anti-RBD and anti-Spike IgG concentrations, with Spearman’s correlation coefficients of 0.83 and 0.84, respectively, while the correlation with anti-N IgG (Rho = 0.46) was low (Figures 5A–5C). As with anti-RBD and anti-Spike binding antibody concentrations, neutralizing antibody activity was significantly higher (*p* < 0.001) in vaccinated compared to nonvaccinated individuals (Figure 5D) and in urban compared to rural populations (Figure 5E).

### Strong preference of antibody binding to Wuhan-Hu-1 CoV-2 compared to Omicron

**Figure 6:**
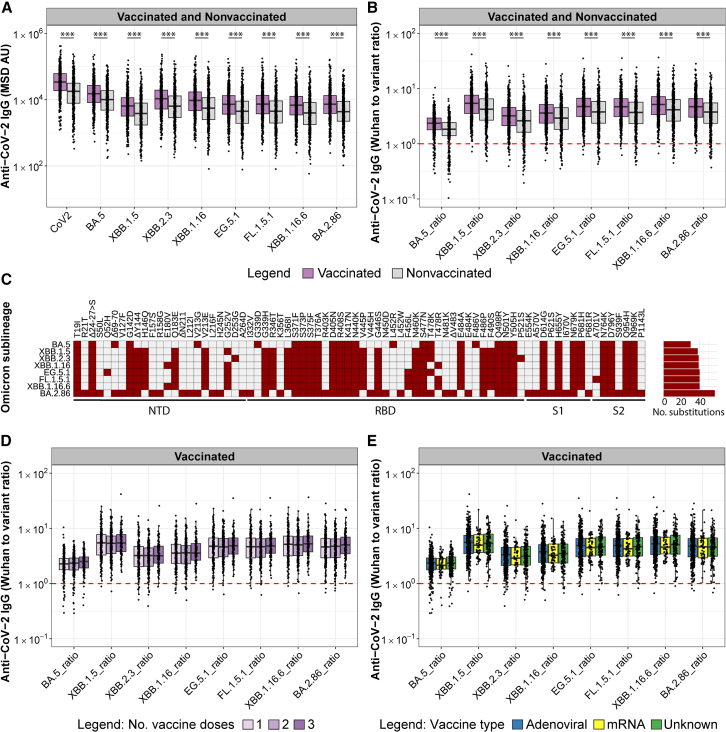
Preference of binding to Wuhan-Hu-1 CoV-2 compared to Omicron (A) Anti-CoV-2 Wuhan-Hu-1 and Omicron sublineage Spike IgG concentrations are shown for vaccinated and nonvaccinated populations. (B) Ratios of anti-Wuhan-Hu-1 to anti-Omicron sublineage Spike concentrations are shown for vaccinated and nonvaccinated populations. The red dashed line indicates a ratio of 1 (equal antibody binding to CoV-2 Wuhan-Hu-1 and Omicron variant Spikes). Significance between vaccinated and nonvaccinated groups was tested with the two-sided Wilcoxon rank-sum test. ∗*p* < 0.05, ∗∗*p* < 0.01, ∗∗∗*p* < 0.001. (C) The number and location of amino acid changes in the Spike protein (NTD, RBD, S1, and S2 regions of Spike) of different Omicron sublineages (mutations according to the MSD V-PLEX COVID-19 Serology Kit insert) compared to Wuhan-Hu-1 CoV-2 are depicted in red. (D) and (E) Ratios of anti-Wuhan-Hu-1 to anti-Omicron sublineage Spike concentrations stratified by the number of vaccine doses (D) and vaccine type (E) received. Significance between groups was tested using the Kruskal-Wallis test. No significant difference was found between groups.

To investigate the impact of exposure to CoV-2 Omicron sublineages (circulating in the months before and at the time of sample collection in 2023) on the antibody responses of the rural and urban study populations, we determined antibody concentrations to Wuhan-Hu-1 and Omicron Spike antigens (Figure 6A) and calculated the ratio of antibody binding to the Wuhan-Hu-1 Spike antigen versus Omicron Spike antigens (Figure 6B). Overall, median antibody concentrations were highest for Wuhan-Hu-1 CoV-2 Spike and decreased for different Omicron sublineages, consistent with the accumulation of amino acid changes (Figure 6C) from BA.5 (2.5-fold decrease in median antibody levels) to XBB.1.16 (4-fold decrease) and BA.2.86 (5.5-fold decrease) (Figure 6A). Considering that BA.2.86 has accumulated a total of 57 amino acid changes, it is notable that IgG binding to this variant was not significantly more impaired than binding to XBB.1.16.6, FL.1.5.1, or EG.5.1, which contain only around 40 amino acid changes. Plotting the ratios of antibody concentrations against Wuhan-Hu-1 divided by Omicron Spike antigens in each individual sample demonstrates a striking preference (ratios >1) for binding to Wuhan-Hu-1 Spike antigens in most of the samples (Figure 6B). Only a few samples showed stronger preference for variant Spike binding (ratios <1), mostly to BA.5. Preferred binding to the Wuhan-Hu-1 Spike antigens was even more pronounced in vaccinated compared to nonvaccinated populations (*p* < 0.001). We did not observe any statistically significant difference in this preference after one, two, or three vaccine doses (Figure 6D), nor following vaccination with either adenoviral or mRNA vaccines (Figure 6E).

## Discussion

As of March 2023, when the Johns Hopkins University stopped updating their online COVID-19 tracking dashboard, a total of 171,229 CoV-2 infections and 1,462 COVID-19-related deaths were reported from Ghana. This corresponds to 60-fold lower case and 84-fold lower death numbers per 100,000 population reported from Ghana compared to the United States in the same time frame (https://coronavirus.jhu.edu/). Our data confirm assumptions on vast underreporting of CoV-2 infections in Ghana. Only four out of the 1,000 study participants had ever been diagnosed with COVID-19, although our serological data indicate very high CoV-2 infection rates, assessed based on an anti-Spike antibody plasma prevalence of 96% in nonvaccinated individuals (99% in vaccinated people). To assess active CoV-2 infection, we utilized an ultrasensitive N antigen detection assay that displayed extremely high diagnostic sensitivity comparable to most PCR-based methods and several hundred-fold more sensitive than most commercial lateral flow assay-based CoV-2 antigen tests.^37^^,^^38^^,^^41^ The detected N antigen saliva prevalence in our study of 1.3% may be an underestimation of active CoV-2 infections in the population, as the study design selected against individuals who felt too sick to participate. Detected active infections combined with the prevalence of the relatively short-lived anti-CoV-2 saliva IgA antibodies, particularly in urban areas, demonstrate high levels of ongoing CoV-2 transmission in Ghana. Our study was not designed to draw conclusions on the severity of detected CoV-2 infections. All individuals with positive N antigen tests however were asymptomatic or presented with only mild symptoms. We can only speculate that underreporting of CoV-2 infections was a result of a high proportion of asymptomatic and mild infections in the Ghanaian population in addition to very limited access of the local population to diagnostic tests.

In Sub-Saharan Africa, over 50% of citizens reside in rural areas,^42^ with limited interaction with travelers or urban communities that could potentially carry CoV-2. Accordingly, we observed significantly higher median anti-CoV-2 IgG antibody concentrations in unvaccinated urban compared to unvaccinated rural populations, suggesting that urban populations had higher levels of exposure to the virus. High anti-CoV-2 Spike antibody positivity of 93% in nonvaccinated rural populations (98% in nonvaccinated urban citizens), however, also demonstrates the striking reach of the virus into remote regions of Ghana. Only modest differences in anti-RBD and anti-Spike IgG antibody concentrations were found in vaccinated urban compared to vaccinated rural populations, indicating that vaccine responses do not seem to be impaired in rural versus urban populations. Saliva IgA concentrations were significantly higher in both unvaccinated and vaccinated urban versus rural participants. Other studies have shown that salivary IgA antibody levels decline rapidly within a few months after CoV-2 infection,^39^^,^^43^ making them an indication of recent infection. We found strongly positive correlations for anti-CoV-2 plasma and saliva IgG concentrations, while plasma IgG and saliva IgA antibody concentrations were less well correlated. This confirms the notion that IgG antibodies mainly enter the mucosa from the blood via transudation, whereas dimeric secretory IgA is produced by local plasma cells.^44^ The stronger correlation of all antibody combinations (plasma vs. saliva IgG, plasma IgG vs. saliva IgA, and saliva IgG vs. IgA) against the RBD and Spike antigens in nonvaccinated compared to vaccinated individuals highlights the fact that systemic and mucosal antibodies are differentially activated in response to infection and vaccination.

Although the World Health Organization declared an end to the COVID-19 public health emergency in May 2023, the virus continues to evolve with new Omicron sublineages on the rise,^45^^,^^46^ posing significant challenges to public health responses. In addition to their fast global spread, these variants have the potential to evade immunity and treatment modalities and eventually require variant-updated COVID-19 vaccine compositions. Genetic analysis of CoV-2 in Africa has shown that infections were predominantly introduced from Europe, with genomes representative of the different CoV-2 clades found on other continents, although genomic under-surveillance has been recognized.^47^ Only about 3% of the reported COVID-19 cases in West Africa have been sequenced from the onset of the pandemic through December 2023. Analysis of the 5,340 CoV-2 sequences from Ghana reveals that several global variants, including Alpha, Beta, Gamma, Delta, Eta, and Kappa, circulated at varying frequencies throughout 2021. Multiple Omicron sublineages emerged as the most prevalent sequences in 2022 and 2023.^36^ Data in this study have shown a striking bias of antibody binding to Wuhan-Hu-1 CoV-2 and up to 6-fold lower antibody concentrations to CoV-2 Omicron variants that have circulated in 2023, suggesting immune imprinting in the Ghanaian population by initial CoV-2 exposures. The concept that “imprinting” by a first viral exposure can shape antibody responses to subsequent variants has been described previously in studies of influenza viruses^48^^,^^49^ and CoV-2.^30^^,^^50^ This phenomenon is likely driven by a faster and higher-affinity memory B cell recall response against conserved epitopes, which outcompetes variant-specific naive B cell responses.^33^ If mutations accumulate in the CoV-2 Spike protein, resulting in a significant loss of conserved epitopes, this could lead to diminished variant cross-neutralization of the immune response. We observed an immune imprinting profile in our study population regardless of the number of vaccine doses or the type of vaccine (mRNA or adenoviral) individuals had received. These data complement reports in other populations showing that individuals who received two or three prior doses of Wuhan-Hu-1 mRNA vaccines exhibited a predominance of imprinted recall responses, limiting new responses to Omicron sublineages.^51^^,^^52^ Furthermore, our data extend these findings, suggesting that similar effects occur in individuals vaccinated with adenoviral vaccines. Other studies have reported that even repeated Omicron exposures after Wuhan-Hu-1 mRNA vaccination could not overcome immune imprinting profiles,^53^^,^^54^ while immune bias was alleviated after repeated Omicron exposures in individuals who had initially received less immunogenic inactivated COVID-19 vaccines.^55^ However, repeated exposure to new antigens, and in particular those with increasing antigenic dissimilarity over time, still broadens responses to new antigens, and regular vaccination with updated CoV-2 vaccines protects against disease and death.^31^^,^^56^^,^^57^ By December 2023, about 37% of the Ghanaian total population were fully vaccinated, with the adenoviral AstraZeneca and Johnson&Johnson vaccines being the most frequently administered (68% of total), followed by Pfizer-BioNTech and Moderna (https://www.ghs.gov.gh/covid19/). Around 20% of the population received a third dose of Wuhan-Hu-1 CoV-2 vaccines. Our data demonstrating high levels of ongoing CoV-2 transmission and significantly reduced recognition of circulating CoV-2 variants in the Ghanaian population calls for the administration of variant-updated vaccines to limit the transmission of the virus and severe disease in vulnerable groups such as elderly individuals and those with comorbidities.

To date, West, Central, and East African countries have largely been omitted from research on the epidemiology of and immune responses to CoV-2 infection. Here, we present the first comprehensive dataset from one of these countries on the prevalence and correlation of anti-CoV-2 plasma and saliva antibodies as well as on antibody responses to Wuhan-Hu-1 CoV-2 versus circulating Omicron sublineages in rural compared to urban areas. Our data provide a snapshot of CoV-2 infection and vaccination prevalence in Ghana at the beginning of the endemic COVID-19 era. In view of high levels of CoV-2 spread in Ghana and reduced recognition of circulating variants, enhanced research efforts are required to shed light on actual severity of ongoing infections and potential beneficial and/or detrimental differences in immune responses to infection and vaccination compared to populations on other continents.

### Limitations of the study

This study is subject to three key limitations. First, we report CoV-2 (sero-)prevalence data from a single time point (December 2023), limiting our ability to draw broader conclusions about the evolution of immunity within the rural and urban populations of Ghana. Second, given the diverse immune histories of CoV-2 infection and vaccination within the general population, we are unable to make definitive conclusions about the separate effects of vaccination versus infection on immune responses. Lastly, our findings are primarily based on antibody binding to different CoV-2 variants, rather than on the neutralization activities against viral variants or antibody effector functions, which would offer a more complete understanding of humoral immunity in the study population. However, we demonstrate a strong correlation between anti-Wuhan-Hu-1 antibody binding and neutralization and provide the first in-depth analysis of antibody profiles in a West African population.

## Resource availability

### Lead contact

Further information and requests for resources and reagents should be directed to and will be fulfilled by the lead contact, Dr. Katharina Röltgen (Katharina.roeltgen@swisstph.ch).

### Materials availability

This study did not generate new unique reagents.

### Data and code availability

The electrochemiluminescence data and neutralization percentages have been deposited on Mendeley at https://doi.org/10.17632/st2xscfb4r.1. This article does not report original code. Any additional information required to reanalyze the data reported in this article is available from the lead contact upon request.

## Acknowledgments

This research was supported by 10.13039/501100001711Swiss National Science Foundation (SNSF) grant PR00P3_208580 to K.R. and a State Secretariat for Education, Research and Innovation (SERI) grant to K.R. We thank all the volunteers for their participation in this study. We also thank Ramon Espejo (MSD) and Natacha Vanattou (MSD) for their kind technical support. The VeroE6/TMPRSS2 cell line was provided by the NIBSC Research Reagent Repository, UK, with thanks to Dr. Makoto Takeda.

## Author contributions

K.R., I.O.D., S.D.B., and D.Y.M. conceptualized the study. B.A.L. built and maintained the REDCap database for the study. E.O.A., I.Q., D.A.O., F.B.A., S.L., D.K.O., C.D., E.D., J.Q., and K.R. contributed to the human sample collection efforts. E.S.L., E.O.A., S.L., J.A.H., A.D., and K.R. contributed to sample processing. M.M.R. and E.S.L. performed the ECL-based laboratory experiments. M.M.R. established and performed the pseudovirus neutralization assays. M.M.R. and K.R. have directly accessed and verified the underlying data reported in the article and analyzed the data. G.B.S. provided materials for the laboratory experiments. K.R. drafted the initial article. M.M.R., E.S.L., G.B.S., S.D.B., and I.O.D. contributed to the interpretation of the results and edited the article for intellectual content. All authors read and approved the final version of the article for submission.

## Declaration of interests

The authors declare no competing interests.

## STAR★Methods

### Key resources table

**Table:** 

REAGENT or RESOURCE	SOURCE	IDENTIFIER
**Antibodies**		

Sulfo-tag conjugated anti-human IgG	Meso Scale Discovery	Cat# D21ADF
Sulfo-tag conjugated anti-human IgA	Meso Scale Discovery	Cat# D21ADE
Reference Standard 1	Meso Scale Discovery	Cat# C00ADK
Biotin SARS-CoV-2 N Antibody	Meso Scale Discovery	Cat# C20AUH
TURBO-BOOST SARS-CoV-2 N Antibody	Meso Scale Discovery	Cat# D20AUH
Human anti-CoV-2 Spike Neutralizing IgG1 Antibody	ACRO Biosystems	Cat# SAD-S35
Mouse anti-VSV-G [8G5F11] IgG2a Antibody	Absolute Antibody	Cat# AB01401–2.0; RRID: AB_2883992

**Bacterial and virus strains**		

VSV-G(ΔG)-Luciferase system	Kerafast	Cat#EH1025-PM

**Biological samples**		

Plasma from 1000 individuals living in rural and urban communities of Ghana	This paper	N/A
Saliva from 1000 individuals living in rural and urban communities of Ghana	This paper	N/A

**Chemicals, peptides, and recombinant proteins**		

MSD GOLD Read Buffer B	Meso Scale Discovery	Cat# R60AM
SARS-CoV-2 N Calibrator	Meso Scale Discovery	Cat# C00ADH
Bright-Glo™ Luciferase Assay System	Promega	Cat# E2620

**Critical commercial assays**		

S-PLEX SARS-CoV-2 N Kit, SECTOR	Meso Scale Discovery	Cat# K150ADHS
V-PLEX COVID-19 Coronavirus Panel 3 (IgG)	Meso Scale Discovery	Cat# K15399U
V-PLEX SARS-CoV-2 Panel 37 (IgG)	Meso Scale Discovery	Cat# K15721U
V-PLEX SARS-CoV-2 Panel 6 (IgA)	Meso Scale Discovery	Cat# K15435U

**Deposited data**		

Electrochemiluminescence data and pseudovirus neutralization percentages	This paper; Mendeley Data	https://doi.org/10.17632/st2xscfb4r.1

**Experimental models: Cell lines**		

HEK293T/17	Cytion	Cat# 305117
VeroE6/TMPRSS2	NIBSC Research Reagent Repository, UK	Cat# 100978

**Recombinant DNA**		

SARS-CoV-2 Spike ORF mammalian expression plasmid	Sino Biological	Cat# VG40589-UT

**Software and algorithms**		

Discovery Workbench Software Version 4.0	Meso Scale Discovery	https://www.mesoscale.com/en/products_and_services/software
R version 4.4.1 base packages	The R Foundation	https://www.rstudio.com/products/rstudio/download/
R version 4.4.1 ggplot2 package	The R Foundation	https://cran.r-project.org/web/packages/ggplot2/index.html

### Experimental model and study participant details

#### Human subjects and biospecimen collection

Demographic information, blood, and saliva was collected for the purpose of this coronavirus epidemiology study in November and December 2023 from 500 volunteers in five rural (Kraboa, Panpanso, Amanfrom, Doblo, and Obom) and 500 volunteers in five urban (Nsawam, Medie, Amasaman, Pokuase, and Ofankor) communities in the Greater Accra and Eastern region of Ghana. The sample size was calculated to detect differences of at least 10% between rural and urban populations (powered at the 80% level with α = 0.05). Teams comprising an interviewer, a phlebotomist, a community volunteer, and a supervisor enrolled the first 100 individuals from each rural and urban community who volunteered to participate in the study, collected the data and samples. All 1,000 participants were asked to provide basic information such as age, sex, and residence, and COVID-19-specific information including COVID-19 vaccination status and type of vaccine (information was recorded based on vaccination card), current symptoms, and history of COVID-19 diagnosis. The cohort included 654 women and 346 men, ranging in age from three to 90 years. Blood was collected from 989 participants in cell preparation tubes (CPT, Becton Dickinson) with sodium citrate and centrifuged at 1,800 x g for 30 min at room temperature. For the collection of saliva individuals were instructed to not eat, drink, smoke or chew gum for at least half an hour before specimen collection. Saliva was collected from 992 participants with SpeciMax saliva collection kits by passive drooling into the provided containers (Thermo Fisher Scientific). On the day of sample collection, plasma and saliva were transported to the laboratory at the Noguchi Memorial Institute for Medical Research (NMIMR), aliquoted and stored at −80°C until they were shipped on dry ice to the Swiss TPH. Approval for the study was obtained from the NMIMR Institutional Review Board (IRB) with federal wide assurance number 00001824, the Ghana Health Service IRB, and the Ethics Committee Northwest and Central Switzerland (EKNZ) with Statement ID AO_2023-00020. All individuals above two years of age were eligible for inclusion in the study. Written informed consent was received from all study participants and in the case of minors from legally authorized representatives (parents or guardians) prior to enrollment.

#### Cell lines

Human embryonic kidney (HEK) 293T/17 cells (Cytion) were cultured in complete Dulbecco’s Modified Eagle’s Medium (DMEM, Sigma-Aldrich) supplemented with 10% fetal bovine serum (FBS), 100 μg/mL of penicillin and 100 μg/mL of streptomycin. Cells were maintained in an incubator at 37°C and 5% CO_2_ following standard cell culture practices. The genetic profile of the cell line was determined by the provider as quality control step to guarantee cell identity.

VeroE6-TMPRSS2 cells (originally isolated from *Chlorocebus* sp.) were purchased from the National Institute for Biological Standards and Control (NIBSC) Research Reagent Repository (UK) but were originally developed by Dr. Makoto Takeda as a modified cell line stably expressing TMPRSS2 in the presence of geneticin (G418). The cells were cultured in complete DMEM and supplemented with 10% FBS and 1 mg/mL of G418 following standard cell culture practices.

The cell lines were not authenticated but were passaged fewer than ten times after thawing from the original vendor stocks. Cell lines were mycoplasma free.

### Method details

#### MSD ECL N antigen detection

Saliva samples were tested for the presence of the CoV-2 N antigen using ECL-based ultrasensitive Meso Scale Discovery (MSD) S-PLEX CoV-2 N kits according to the manufacturer’s instructions. Briefly, saliva samples were tested at a 1:2 dilution in MSD blocking solution on S-PLEX 96-well SECTOR plates coated with Biotin CoV-2 N capture antibody. A 7-point calibration curve using CoV-2 N Calibrator and a blank well were run in duplicate on each plate. The N antigen was detected with MSD TURBO-BOOST CoV-2 N detection antibodies. A signal enhancement step was performed using the MSD S-PLEX Enhance solution and TURBO-TAG detection solution. Plates were read after addition of MSD GOLD Read Buffer B using a MESO QuickPlex SQ 120 instrument.

#### Serological assays

Plasma and saliva samples were tested for the presence of anti-CoV-2 antibodies using multiplexed ECL detection in a 96-well plate format with MSD V-PLEX serology panels and instrumentation according to the manufacturer’s instructions. V-PLEX COVID-19 Coronavirus Panel 3 kits were used to detect IgG antibody concentrations to the Wuhan-Hu-1 CoV-2 N, RBD, and Spike antigens. V-PLEX CoV-2 Panel 37 kits were used to determine IgG antibody concentrations to the Spike antigens of different CoV-2 Omicron sublineages, including BA.2.86, BA.5, EG.5.1, FL.1.5.1, XBB.1.5, XBB.1.16, XBB.1.16.6, and XBB.2.3. V-PLEX CoV-2 Panel 6 kits were used to determine IgA antibody concentrations to the Wuhan-Hu-1 CoV-2 Spike, RBD, and N antigens. Plasma samples were analyzed at a 1:5,000 dilution in MSD Diluent 100, while saliva samples were analyzed at a 1:5 dilution for IgG and at a 1:20 dilution for IgA in MSD Diluent 2. Plasma and saliva antibodies were detected with anti-human IgG or IgA antibodies conjugated to SULFO-TAG ECL labels and read with a MESO QuickPlex SQ 120 instrument after addition of MSD GOLD Read Buffer B. Each plate contained duplicates of a 7-point calibration curve with serial dilution of a Reference Standard, a blank well and three positive control samples. Calibration curves were used to calculate antibody unit concentrations (arbitrary units, AU) by back-fitting ECL signals measured for each test sample to the curve. Cutoff values for positive plasma antibody test results for the Wuhan-Hu-1 CoV-2 N, RBD, and Spike antigens were determined by analyzing 400 plasma samples collected in 2013 from residents of the Ashanti and Central Region of Ghana,^58^ neighboring the Eastern and GAR Regions of this study. Cutoff values for seroconversion were calculated by adding three standard deviations to mean AU values. The cutoff value for Spike (1,535 AU/mL) determined in this study was similar to the one identified by the manufacturer (1,960 AU/mL) based on pre-pandemic and COVID-19 patient samples from the U.S. Cutoff values for RBD (1,573 AU/mL) and N (13,408 AU/mL) determined in this study were about 3-fold higher than the ones identified by the manufacturer (538 AU/mL for RBD and 5,000 AU/mL for N), but still similar considering the broad dynamic range of the ECL assays.

#### CoV-2 S pseudotyped virus production and neutralization assay

The VSV-G(ΔG)-Luciferase (Kerafast) producer virus was propagated using HEK293T/17 cells (Cytion) according to the original protocol described in.^59^ The CoV-2 Spike pseudotyped virus was generated by transfecting HEK293T/17 cells with the plasmid for the expression of the CoV-2 Spike protein with reference sequence YP_009724390.1 (Sino Biologicals). 24 h later the cells were transduced with the VSV-G(ΔG)-Luciferase producer virus using a 0.2 multiplicity of infection (MOI) for 2 h in cell growth conditions to allow virus adsorption. Then, the cells were gently washed with PBS and fresh DMEM containing 5% FBS and 100 U/mL of penicillin/streptomycin were added. The pseudotyped virus was collected 24 h after transduction and cleared from cell debris by centrifugation for 10 min at 500 x g and then by filtering through a 0.45 μm filter. Plasma samples were tested in the *in vitro* neutralization assay by diluting them to 1:400 in cell culture media and incubating them for 1 h at 37°C with the CoV-2 Spike pseudotyped virus with a MOI of 0.01 including 0.5 μg/mL of mouse anti-VSV-G antibody to obliterate unspecific signal from the producer virus. Human anti-CoV-2 Spike neutralizing antibody concentrations of 1, 0.5, and 0.25 μg/mL were used as neutralization controls. The infection was performed in VeroE6/TMPRSS2 cells for 20 h followed by removal of media and addition of the Bright-Glo Luciferase Assay System (Promega). Luminescence was detected after 5 min using the Spark Microplate Reader (Tecan).

### Quantification and statistical analysis

Data were illustrated using the R ggplot2 package. Box-whisker plots show median (horizontal line), interquartile range (box), and the end of the low whisker representing the smallest observation greater than or equal to the 25% quantile minus 1.5 times the interquartile range, and the end of the upper whisker representing the largest observation less than or equal to the 75% quantile plus 1.5 times the interquartile range. Ratios of concentrations of IgG binding to Wuhan-Hu-1 Spike compared to Omicron Spikes were calculated to quantify serological binding preference for these antigens. A ratio of one indicates even preference, while ratios greater than one indicate preferential binding of Wuhan-Hu-1 over viral variant antigens and ratios lower than one indicate preferential binding of viral variant antigens over Wuhan-Hu-1 CoV-2 Spike.

Statistical analyses were conducted using R base packages. To determine significance between two groups (vaccinated versus nonvaccinated, rural versus urban, male versus female) we employed the two-sided Wilcoxon rank-sum test. For comparison between several groups (number of vaccine doses, vaccine type) we used the Kruskal-Wallis test. Spearman rank correlation was used to assess the strength of associations between two different variables. Viral neutralization percentages were calculated using the formula:

Neutralization%=100−[(“samplesignal”−“backgroundsignal”)÷(“maximalsignal”−“backgroundsignal”)×100],

where the background signal represents the measurement from a well containing neither antibody nor virus and maximal signal corresponds to the measurement from a well with viral infection in the absence of any antibody.

## References

[1] Lewis H.C., Ware H., Whelan M., Subissi L., Li Z., Ma X., Nardone A., Valenciano M., Cheng B., Noel K. (2022). SARS-CoV-2 infection in Africa: a systematic review and meta-analysis of standardised seroprevalence studies, from January 2020 to December 2021. BMJ Glob. Health.

[2] Bergeri I., Whelan M.G., Ware H., Subissi L., Nardone A., Lewis H.C., Li Z., Ma X., Valenciano M., Cheng B. (2022). Global SARS-CoV-2 seroprevalence from January 2020 to April 2022: A systematic review and meta-analysis of standardized population-based studies. PLoS Med..

[3] Msemburi W., Karlinsky A., Knutson V., Aleshin-Guendel S., Chatterji S., Wakefield J. (2023). The WHO estimates of excess mortality associated with the COVID-19 pandemic. Nature.

[4] Moeti M., Makubalo L., Gueye A.S., Balde T., Karamagi H., Awandare G., Thumbi S.M., Zhang F., Mutapi F., Woolhouse M. (2023). Conflicting COVID-19 excess mortality estimates. Lancet.

[5] McKay T., Robinson R.S., Musungu S., Padi-Adjirackor N.A., Angotti N. (2024). The Missing Millions: Uncovering the Burden of Covid-19 Cases and Deaths in the African Region. Popul. Dev. Rev..

[6] van Dorst M.M.A.R., Pyuza J.J., Nkurunungi G., Kullaya V.I., Smits H.H., Hogendoorn P.C.W., Wammes L.J., Everts B., Elliott A.M., Jochems S.P., Yazdanbakhsh M. (2024). Immunological factors linked to geographical variation in vaccine responses. Nat. Rev. Immunol..

[7] Lynn D.J., Benson S.C., Lynn M.A., Pulendran B. (2022). Modulation of immune responses to vaccination by the microbiota: implications and potential mechanisms. Nat. Rev. Immunol..

[8] Netea M.G., Domínguez-Andrés J., van de Veerdonk F.L., van Crevel R., Pulendran B., van der Meer J.W.M. (2022). Natural resistance against infections: focus on COVID-19. Trends Immunol..

[9] Jiang V., Jiang B., Tate J., Parashar U.D., Patel M.M. (2010). Performance of rotavirus vaccines in developed and developing countries. Hum. Vaccin..

[10] Jongo S.A., Shekalaghe S.A., Church L.W.P., Ruben A.J., Schindler T., Zenklusen I., Rutishauser T., Rothen J., Tumbo A., Mkindi C. (2018). Safety, Immunogenicity, and Protective Efficacy against Controlled Human Malaria Infection of Plasmodium falciparum Sporozoite Vaccine in Tanzanian Adults. Am. J. Trop. Med. Hyg..

[11] Muyanja E., Ssemaganda A., Ngauv P., Cubas R., Perrin H., Srinivasan D., Canderan G., Lawson B., Kopycinski J., Graham A.S. (2014). Immune activation alters cellular and humoral responses to yellow fever 17D vaccine. J. Clin. Investig..

[12] van Riet E., Adegnika A.A., Retra K., Vieira R., Tielens A.G.M., Lell B., Issifou S., Hartgers F.C., Rimmelzwaan G.F., Kremsner P.G., Yazdanbakhsh M. (2007). Cellular and humoral responses to influenza in gabonese children living in rural and semi-urban areas. J. Infect. Dis..

[13] Kabagenyi J., Natukunda A., Nassuuna J., Sanya R.E., Nampijja M., Webb E.L., Elliott A.M., Nkurunungi G. (2020). Urban-rural differences in immune responses to mycobacterial and tetanus vaccine antigens in a tropical setting: A role for helminths?. Parasitol. Int..

[14] Röltgen K., Boyd S.D. (2023). Antibody and B Cell Responses to SARS-CoV-2 Infection and Vaccination: The End of the Beginning. Annu. Rev. Pathol..

[15] Sette A., Crotty S. (2021). Adaptive immunity to SARS-CoV-2 and COVID-19. Cell.

[16] Röltgen K., Powell A.E., Wirz O.F., Stevens B.A., Hogan C.A., Najeeb J., Hunter M., Wang H., Sahoo M.K., Huang C. (2020). Defining the features and duration of antibody responses to SARS-CoV-2 infection associated with disease severity and outcome. Sci. Immunol..

[17] Wajnberg A., Mansour M., Leven E., Bouvier N.M., Patel G., Firpo-Betancourt A., Mendu R., Jhang J., Arinsburg S., Gitman M. (2020). Humoral response and PCR positivity in patients with COVID-19 in the New York City region, USA: an observational study. Lancet. Microbe.

[18] Long Q.X., Liu B.Z., Deng H.J., Wu G.C., Deng K., Chen Y.K., Liao P., Qiu J.F., Lin Y., Cai X.F. (2020). Antibody responses to SARS-CoV-2 in patients with COVID-19. Nat. Med..

[19] Jackson C.B., Farzan M., Chen B., Choe H. (2022). Mechanisms of SARS-CoV-2 entry into cells. Nat. Rev. Mol. Cell Biol..

[20] Baden L.R., El Sahly H.M., Essink B., Kotloff K., Frey S., Novak R., Diemert D., Spector S.A., Rouphael N., Creech C.B. (2021). Efficacy and Safety of the mRNA-1273 SARS-CoV-2 Vaccine. N. Engl. J. Med..

[21] Polack F.P., Thomas S.J., Kitchin N., Absalon J., Gurtman A., Lockhart S., Perez J.L., Pérez Marc G., Moreira E.D., Zerbini C. (2020). Safety and Efficacy of the BNT162b2 mRNA Covid-19 Vaccine. N. Engl. J. Med..

[22] Voysey M., Clemens S.A.C., Madhi S.A., Weckx L.Y., Folegatti P.M., Aley P.K., Angus B., Baillie V.L., Barnabas S.L., Bhorat Q.E. (2021). Safety and efficacy of the ChAdOx1 nCoV-19 vaccine (AZD1222) against SARS-CoV-2: an interim analysis of four randomised controlled trials in Brazil, South Africa, and the UK. Lancet.

[23] Sadoff J., Gray G., Vandebosch A., Cárdenas V., Shukarev G., Grinsztejn B., Goepfert P.A., Truyers C., Fennema H., Spiessens B. (2021). Safety and Efficacy of Single-Dose Ad26.COV2.S Vaccine against Covid-19. N. Engl. J. Med..

[24] Feng S., Phillips D.J., White T., Sayal H., Aley P.K., Bibi S., Dold C., Fuskova M., Gilbert S.C., Hirsch I. (2021). Correlates of protection against symptomatic and asymptomatic SARS-CoV-2 infection. Nat. Med..

[25] Khoury D.S., Cromer D., Reynaldi A., Schlub T.E., Wheatley A.K., Juno J.A., Subbarao K., Kent S.J., Triccas J.A., Davenport M.P. (2021). Neutralizing antibody levels are highly predictive of immune protection from symptomatic SARS-CoV-2 infection. Nat. Med..

[26] Gilbert P.B., Montefiori D.C., McDermott A.B., Fong Y., Benkeser D., Deng W., Zhou H., Houchens C.R., Martins K., Jayashankar L. (2022). Immune correlates analysis of the mRNA-1273 COVID-19 vaccine efficacy clinical trial. Science.

[27] Carabelli A.M., Peacock T.P., Thorne L.G., Harvey W.T., Hughes J., Peacock S.J., Barclay W.S., de Silva T.I., Towers G.J., Robertson D.L., COVID-19 Genomics UK Consortium (2023). SARS-CoV-2 variant biology: immune escape, transmission and fitness. Nat. Rev. Microbiol..

[28] Willett B.J., Grove J., MacLean O.A., Wilkie C., De Lorenzo G., Furnon W., Cantoni D., Scott S., Logan N., Ashraf S. (2022). SARS-CoV-2 Omicron is an immune escape variant with an altered cell entry pathway. Nat. Microbiol..

[29] Uraki R., Ito M., Furusawa Y., Yamayoshi S., Iwatsuki-Horimoto K., Adachi E., Saito M., Koga M., Tsutsumi T., Yamamoto S. (2023). Humoral immune evasion of the omicron subvariants BQ.1.1 and XBB. Lancet Infect. Dis..

[30] Röltgen K., Nielsen S.C.A., Silva O., Younes S.F., Zaslavsky M., Costales C., Yang F., Wirz O.F., Solis D., Hoh R.A. (2022). Immune imprinting, breadth of variant recognition, and germinal center response in human SARS-CoV-2 infection and vaccination. Cell.

[31] Cobey S. (2024). Vaccination against rapidly evolving pathogens and the entanglements of memory. Nat. Immunol..

[32] Koutsakos M., Ellebedy A.H. (2023). Immunological imprinting: Understanding COVID-19. Immunity.

[33] King S.M., Bryan S.P., Hilchey S.P., Wang J., Zand M.S. (2023). First Impressions Matter: Immune Imprinting and Antibody Cross-Reactivity in Influenza and SARS-CoV-2. Pathogens.

[34] Sangster M.Y., Nguyen P.Q.T., Topham D.J. (2019). Role of Memory B Cells in Hemagglutinin-Specific Antibody Production Following Human Influenza A Virus Infection. Pathogens.

[35] Andrews S.F., Chambers M.J., Schramm C.A., Plyler J., Raab J.E., Kanekiyo M., Gillespie R.A., Ransier A., Darko S., Hu J. (2019). Activation Dynamics and Immunoglobulin Evolution of Pre-existing and Newly Generated Human Memory B cell Responses to Influenza Hemagglutinin. Immunity.

[36] Togo J., Somboro A.M., Dolo O., Traore F.T., Guindo I., Fofana D.B., Todesco E., Marcelin A.G., Calvez V., Holl J. (2024). Dynamics of SARS-CoV-2 variants in West Africa: Insights into genomic surveillance in resource-constrained settings. Infect. Genet. Evol..

[37] Lippi G., Henry B.M., Montagnana M., Plebani M. (2022). Diagnostic accuracy of the ultrasensitive S-PLEX SARS-CoV-2 N electrochemiluminescence immunoassay. Clin. Chem. Lab. Med..

[38] Ren A., Sohaei D., Ulndreaj A., Pons-Belda O.D., Fernandez-Uriarte A., Zacharioudakis I., Sigal G.B., Stengelin M., Mathew A., Campbell C. (2022). Ultrasensitive assay for saliva-based SARS-CoV-2 antigen detection. Clin. Chem. Lab. Med..

[39] Isho B., Abe K.T., Zuo M., Jamal A.J., Rathod B., Wang J.H., Li Z., Chao G., Rojas O.L., Bang Y.M. (2020). Persistence of serum and saliva antibody responses to SARS-CoV-2 spike antigens in COVID-19 patients. Sci. Immunol..

[40] Alkharaan H., Bayati S., Hellström C., Aleman S., Olsson A., Lindahl K., Bogdanovic G., Healy K., Tsilingaridis G., De Palma P. (2021). Persisting Salivary IgG Against SARS-CoV-2 at 9 Months After Mild COVID-19: A Complementary Approach to Population Surveys. J. Infect. Dis..

[41] Pollock Nira R., Savage Timothy J., Wardell H., Lee Rose A., Mathew A., Stengelin M., Sigal George B. (2021). Correlation of SARS-CoV-2 Nucleocapsid Antigen and RNA Concentrations in Nasopharyngeal Samples from Children and Adults Using an Ultrasensitive and Quantitative Antigen Assay. J. Clin. Microbiol..

[42] Gutu Sakketa T. (2023). Urbanisation and rural development in sub-Saharan Africa: A review of pathways and impacts. Res. Global..

[43] Sterlin D., Mathian A., Miyara M., Mohr A., Anna F., Claër L., Quentric P., Fadlallah J., Devilliers H., Ghillani P. (2021). IgA dominates the early neutralizing antibody response to SARS-CoV-2. Sci. Transl. Med..

[44] Bemark M., Angeletti D. (2021). Know your enemy or find your friend?-Induction of IgA at mucosal surfaces. Immunol. Rev..

[45] Kaku Y., Yo M.S., Tolentino J.E., Uriu K., Okumura K., Ito J., Sato K., Genotype to Phenotype Japan G2P-Japan Consortium (2024). Virological characteristics of the SARS-CoV-2 KP.3, LB.1, and KP.2.3 variants. Lancet Infect. Dis..

[46] Planas D., Staropoli I., Michel V., Lemoine F., Donati F., Prot M., Porrot F., Guivel-Benhassine F., Jeyarajah B., Brisebarre A. (2024). Distinct evolution of SARS-CoV-2 Omicron XBB and BA.2.86/JN.1 lineages combining increased fitness and antibody evasion. Nat. Commun..

[47] Okoh O.S., Nii-Trebi N.I., Jakkari A., Olaniran T.T., Senbadejo T.Y., Kafintu-kwashie A.A., Dairo E.O., Ganiyu T.O., Akaninyene I.E., Ezediuno L.O. (2022). Epidemiology and genetic diversity of SARS-CoV-2 lineages circulating in Africa. iScience.

[48] Gostic K.M., Ambrose M., Worobey M., Lloyd-Smith J.O. (2016). Potent protection against H5N1 and H7N9 influenza via childhood hemagglutinin imprinting. Science.

[49] Edler P., Schwab L.S.U., Aban M., Wille M., Spirason N., Deng Y.M., Carlock M.A., Ross T.M., Juno J.A., Rockman S. (2024). Immune imprinting in early life shapes cross-reactivity to influenza B virus haemagglutinin. Nat. Microbiol..

[50] Tortorici M.A., Addetia A., Seo A.J., Brown J., Sprouse K., Logue J., Clark E., Franko N., Chu H., Veesler D. (2024). Persistent immune imprinting occurs after vaccination with the COVID-19 XBB.1.5 mRNA booster in humans. Immunity.

[51] Quandt J., Muik A., Salisch N., Lui B.G., Lutz S., Krüger K., Wallisch A.-K., Adams-Quack P., Bacher M., Finlayson A. (2022). Omicron BA.1 breakthrough infection drives cross-variant neutralization and memory B cell formation against conserved epitopes. Sci. Immunol..

[52] Pušnik J., Zorn J., Monzon-Posadas W.O., Peters K., Osypchuk E., Blaschke S., Streeck H. (2024). Vaccination impairs de novo immune response to omicron breakthrough infection, a precondition for the original antigenic sin. Nat. Commun..

[53] Muik A., Quandt J., Lui B.G., Bacher M., Lutz S., Grünenthal M., Toker A., Grosser J., Ozhelvaci O., Blokhina O. (2024). Immunity against conserved epitopes dominates after two consecutive exposures to SARS-CoV-2 Omicron BA.1. Cell Rep..

[54] Johnston T.S., Li S.H., Painter M.M., Atkinson R.K., Douek N.R., Reeg D.B., Douek D.C., Wherry E.J., Hensley S.E. (2024). Immunological imprinting shapes the specificity of human antibody responses against SARS-CoV-2 variants. Immunity.

[55] Yisimayi A., Song W., Wang J., Jian F., Yu Y., Chen X., Xu Y., Yang S., Niu X., Xiao T. (2024). Repeated Omicron exposures override ancestral SARS-CoV-2 immune imprinting. Nature.

[56] Tseng H.F., Ackerson B.K., Sy L.S., Tubert J.E., Luo Y., Qiu S., Lee G.S., Bruxvoort K.J., Ku J.H., Florea A. (2023). mRNA-1273 bivalent (original and Omicron) COVID-19 vaccine effectiveness against COVID-19 outcomes in the United States. Nat. Commun..

[57] Lin D.Y., Du Y., Xu Y., Paritala S., Donahue M., Maloney P. (2024). Durability of XBB.1.5 Vaccines against Omicron Subvariants. N. Engl. J. Med..

[58] Ampah K.A., Nickel B., Asare P., Ross A., De-Graft D., Kerber S., Spallek R., Singh M., Pluschke G., Yeboah-Manu D., Röltgen K. (2016). A Sero-epidemiological Approach to Explore Transmission of Mycobacterium ulcerans. PLoS Negl. Trop. Dis..

[59] Whitt M.A. (2010). Generation of VSV pseudotypes using recombinant ΔG-VSV for studies on virus entry, identification of entry inhibitors, and immune responses to vaccines. J. Virol. Methods.

