# Supplementary material for: Immune imprinting and antibody profiles to SARS-CoV-2 in urban and rural Ghana
Source: iScience. 2025 Apr 23;28(5):112511. doi: 10.1016/j.isci.2025.112511 (PMC12135443; doi:10.1016/j.isci.2025.112511)
Supplement: Document S1. Figures S1–S3 [file mmc1.pdf]

iScience, Volume *28*

Supplemental information

Immune imprinting and antibody profiles to SARS-CoV-2 in urban and rural Ghana

Martin Montiel-Ruiz, Elvis S. Lomotey, Elizabeth Obeng-Aboagye, Isaac Quaye, Daniel A. Odumang, Florence B. Amakye, Bernard A. Logonia, Salomé Lochmann, Joseph A. Hayford, Dickson K. Osabutey, Angelica Daakyire, Christopher Dorcoo, Edward Dumashie, Joseph Quartey, Dorothy Yeboah-Manu, George B. Sigal, Scott D. Boyd, Irene Owusu Donkor, and Katharina Röltgen

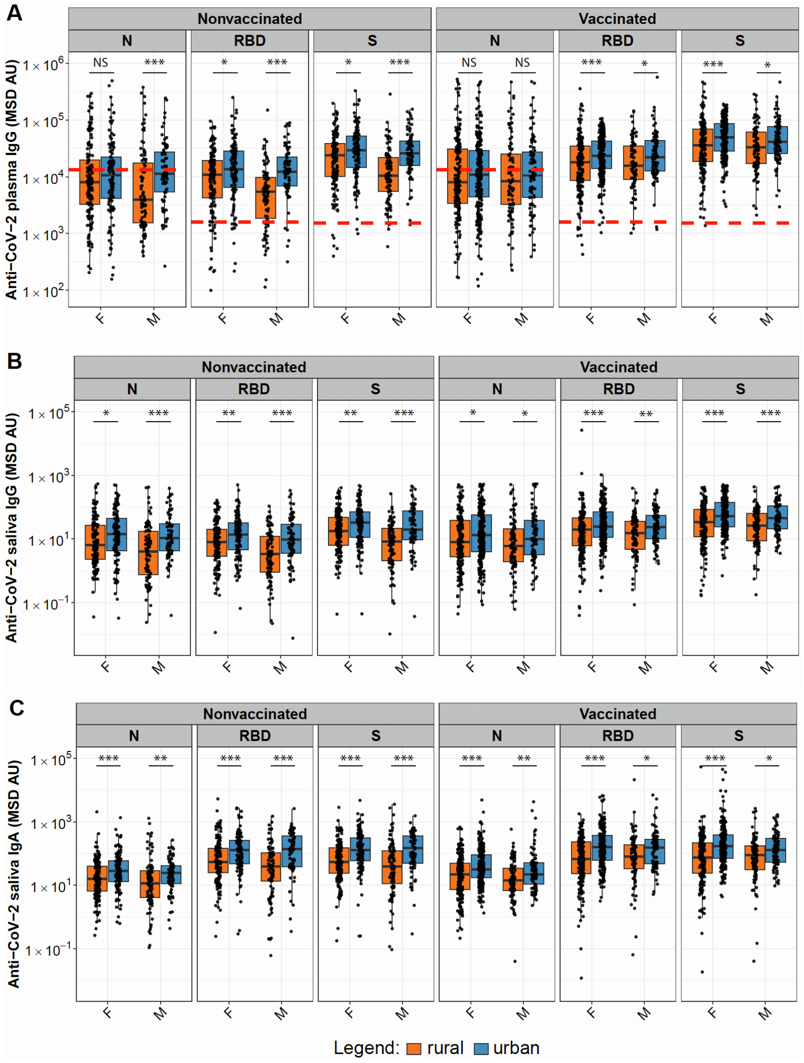

Figure S1. Exposure of rural and urban populations to CoV-2 by sex.

Anti-N, anti-RBD, and anti-S plasma IgG (A), saliva IgG (B), and saliva IgA (C) antibody concentrations in MSD Arbitrary Units (AU) are shown for non-vaccinated and vaccinated rural (orange) and urban (blue) populations, categorized by sex. Box-whisker plots show the median and interquartile range as the box and the whisker ends as the most extreme values within 1.5 times the interquartile range below the 25% quantile and above the 75% quantile. Red dashed lines indicate the cutoff values for the positivity of each assay.

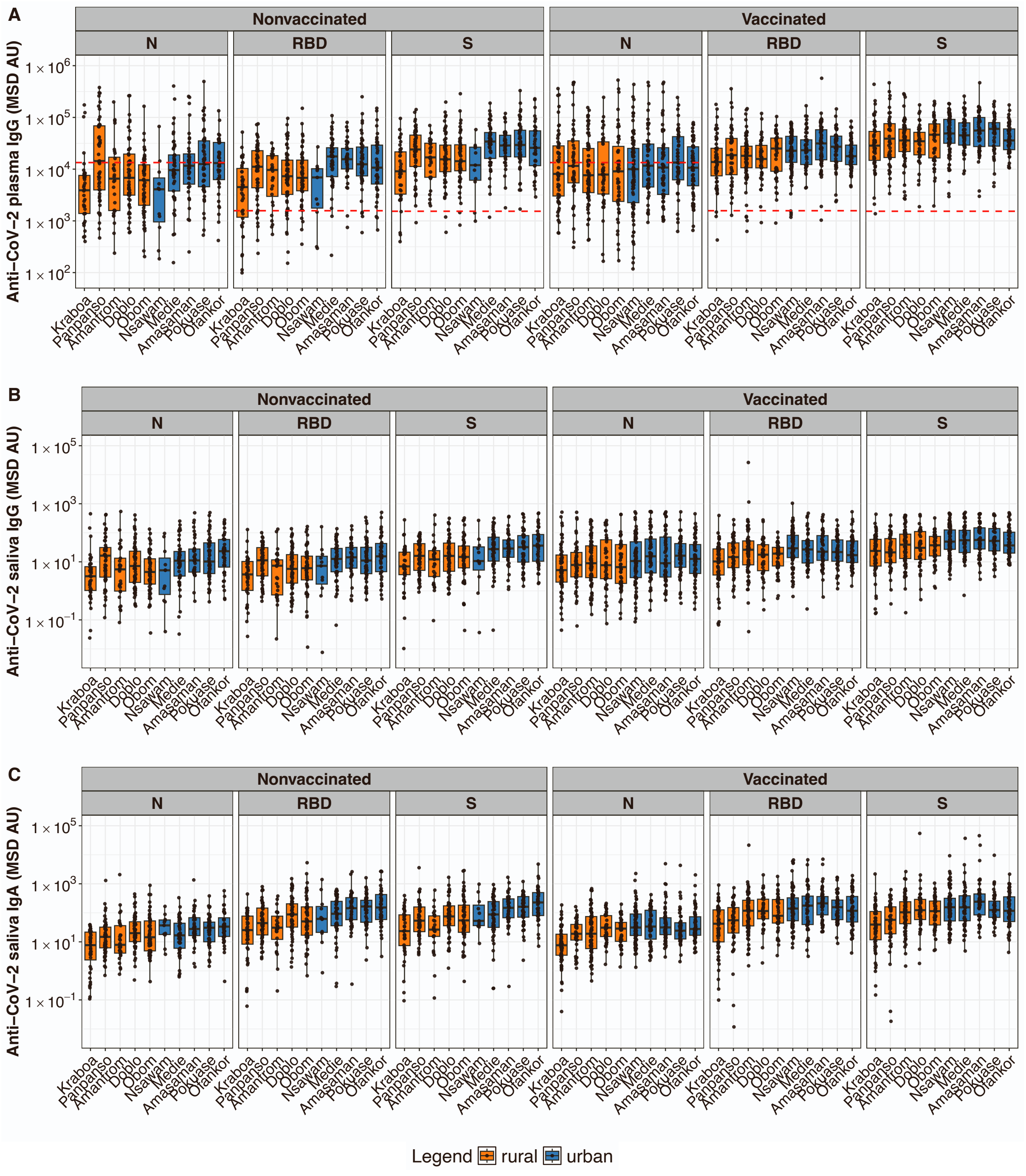

Figure S2. Exposure of rural and urban populations to CoV-2 by community.

Anti-N, anti-RBD, and anti-S plasma IgG (A), saliva IgG (B), and saliva IgA (C) antibody concentrations in MSD Arbitrary Units (AU) are shown for non-vaccinated and vaccinated rural (orange) and urban (blue) populations across communities. Box-whisker plots show the median and interquartile range as the box and the whisker ends as the most extreme values within 1.5 times the interquartile range below the 25% quantile and above the 75% quantile. Red dashed lines indicate the cutoff values for the positivity of each assay.

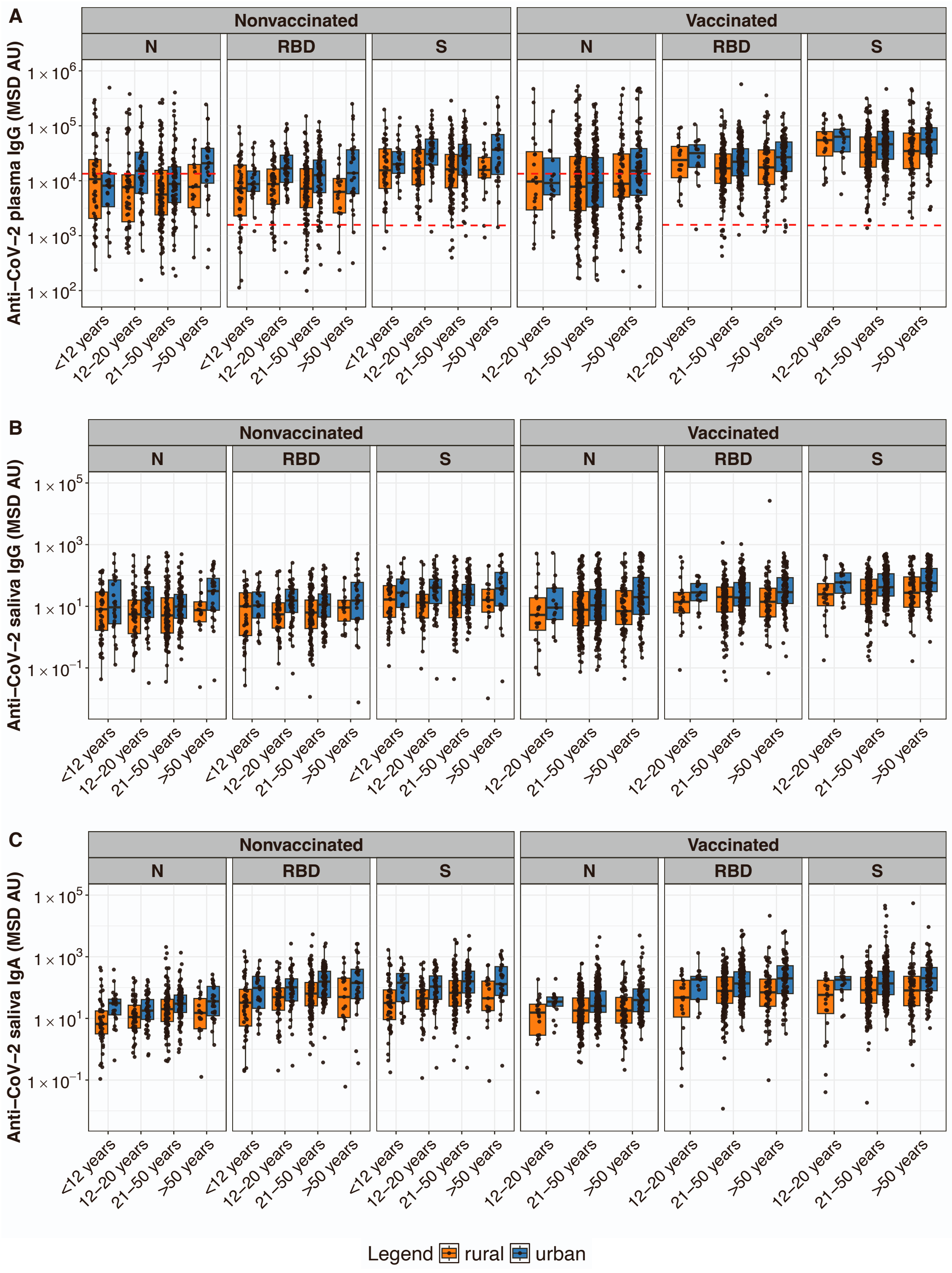

Figure S3. Exposure of rural and urban populations to CoV-2 by age.

Anti-N, anti-RBD, and anti-S plasma IgG (A), saliva IgG (B), and saliva IgA (C) antibody concentrations in MSD Arbitrary Units (AU) are shown for non-vaccinated and vaccinated rural (orange) and urban (blue) populations across age groups. Box-whisker plots show the median and interquartile range as the box and the whisker ends as the most extreme values within 1.5 times the interquartile range below the 25% quantile and above the 75% quantile. Red dashed lines indicate the cutoff values for the positivity of each assay.
